# More subjects are required for ventrolateral than dorsolateral prefrontal TMS because of intolerability and potential drop-out

**DOI:** 10.1371/journal.pone.0217826

**Published:** 2019-06-03

**Authors:** Shuyan Han, Akitoshi Ogawa, Takahiro Osada, Akimitsu Suda, Masaki Tanaka, Hitoshi Nanjo, Yasushi Shimo, Nobutaka Hattori, Seiki Konishi

**Affiliations:** 1 Department of Neurophysiology, Juntendo University School of Medicine, Tokyo, Japan; 2 Department of Neurology, Juntendo University School of Medicine, Tokyo, Japan; 3 Research Institute for Diseases of Old Age, Juntendo University School of Medicine, Tokyo, Japan; 4 Sportology Center, Juntendo University School of Medicine, Tokyo, Japan; 5 Advanced Research Institute for Health Science, Juntendo University School of Medicine, Tokyo, Japan; University of Ottawa, CANADA

## Abstract

Transcranial magnetic stimulation (TMS) of the human lateral prefrontal cortex, particularly the ventral region, often causes considerable discomfort to subjects. To date, in contrast to abundant literature on stimulations to the dorsolateral prefrontal cortex, the ventrolateral prefrontal cortex has been less frequently stimulated, partly because some subjects are intolerable of stimulation to the ventrolateral prefrontal cortex. To predict the additional number of subjects required for the stimulation of the dorsolateral and ventrolateral prefrontal cortices, 20 young healthy subjects reported two evaluation scores: the discomfort caused by TMS and the resulting intolerability to complete the TMS experiments. Single-pulse stimulation (SPS) or theta-burst stimulation (TBS) was administered to the lateral prefrontal cortex. The high-resolution extended 10–20 system was used to provide accurate estimation of the voxelwise scores. The discomfort ratings with the SPS and TBS were relatively higher in the ventrolateral prefrontal cortex than those in the dorsolateral prefrontal cortex. Both the SPS and TBS elicited maximal discomfort at the stimulation position F8. The SPS and TBS to F8 under the standard TMS protocols were intolerable for approximately one half (11 and 10, respectively) of the subjects. The intolerability was further calculated for all voxels in the lateral prefrontal cortex, which enabled us to estimate the additional number of subjects required for specific target areas. These results suggest that prior knowledge of subjects’ discomfort during stimulation of the lateral prefrontal cortex can be of practical use in the experimental planning of the appropriate number of recruited subjects and provide the database for the probability of intolerability that can be used to predict the additional number of subjects.

## Introduction

Transcranial magnetic stimulation (TMS) of the lateral prefrontal cortex can be powerful in noninvasive studies of the causal relationship of brain regions with cognitive functions [1–8]. However, TMS research in the lateral prefrontal cortex is limited. Stimulation to the lateral prefrontal cortex often causes considerable discomfort to subjects [9–16] due to a greater density of muscles and nerves in the prefrontal regions [17–19]. Stimulation of the dorsal region of the lateral prefrontal cortex has frequently been reported [20–40], while fewer (approximately one-third, based on our PubMed search) studies have reported stimulation of the ventral region of the lateral prefrontal cortex [41–48]. Stimulation of brain regions that tend to elicit discomfort appears nonpreferred, partly because some subjects are intolerant of stimulation to the brain regions. The intolerability of unpreferred brain region stimulation results in data loss of some subjects and, therefore, the loss of study power. However, prior knowledge of intolerability caused by stimulation is helpful in the experimental planning of the appropriate sample size.

Previous studies have performed detailed scalp mapping of discomfort using the standard 10–20 system during single-pulse stimulation (SPS) and have provided evidence that discomfort exerts small but significant delays in the reaction times of cognitive tasks [49, 50]. We extended the prior studies such that estimation of the additional number of subjects to be recruited in stimulating the lateral prefrontal cortex could be provided. Specifically, in the present study, subjects reported discomfort caused by TMS and the resulting intolerability to complete TMS experiments. Discomfort is a subjective feeling that makes an experiment difficult to continue, and intolerability is a result of discomfort that can be binarized regarding whether or not the experiment can be continued. In addition to SPS, which is the most basic stimulation, we also examined continuous theta-burst stimulation (TBS) [2, 51], which seems to be the most useful rTMS in studying cognitive functions because of the prevalence and reasonable duration of effects. Moreover, the high-resolution extended 10–20 system (10–10 system) [52, 53] was applied to the lateral prefrontal cortex, which can be used to estimate the discomfort and intolerability of stimulation of any region in the lateral prefrontal cortex.

## Materials and methods

### Subjects

Twenty right-handed subjects [10 men and 10 women; age: 26.0 ± 9.0 years (mean ± SD), ranging from 20 to 49] participated in the study. At least 20 samples are known to be required to minimize false positive observations [54]. Written informed consent was obtained from all subjects based on the guidelines of the Declaration of Helsinki. The experimental procedures were approved by the Institutional Review Board of Juntendo University School of Medicine. All raw data are uploaded as supplementary materials (S1 Dataset).

### TMS procedures

**Fig 1:**
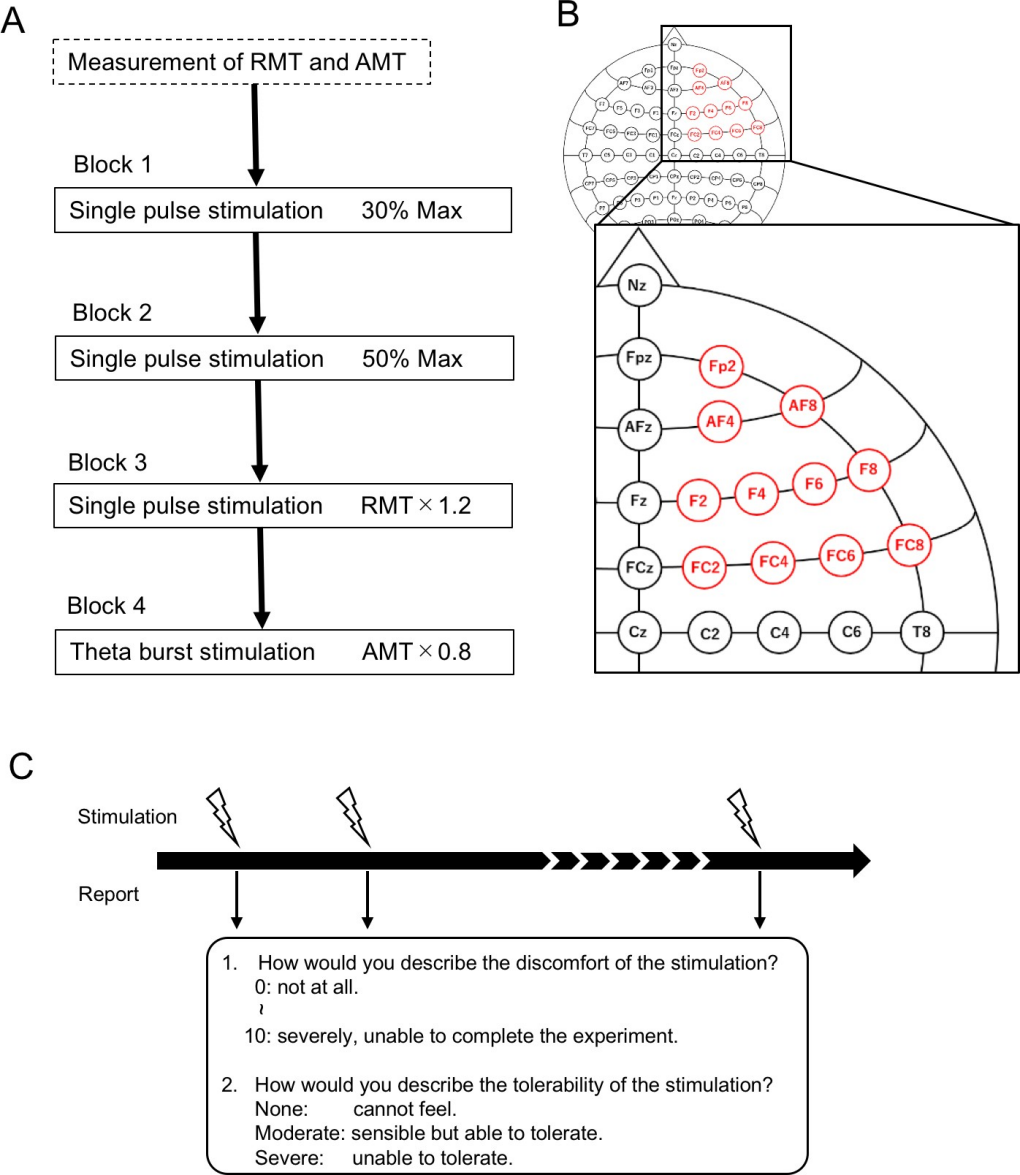
Experimental protocols of the TMS study. (A) Four TMS stimulation conditions that were administered to subjects. After the measurement of the RMT and AMT, subjects received four types of stimulations in separate blocks. (B) High-resolution extended 10–20 system (11 stimulation positions) that covered the lateral prefrontal cortex. (C) Evaluation scores of discomfort and intolerability reported after each trial at each stimulation position. Discomfort was rated from 0 to 10, and intolerability was defined as “none,” “moderate” and “severe”.

TMS was administered using a hand-held figure-of-eight coil (7-cm diameter at each wing; The Magstim Company, Whitland, Dyfed, UK). Monophasic SPS and biphasic TBS [2, 51] were administered in the main experiments (Blocks 1 to 5) (Fig 1A). Prior to the main experiments, monophasic SPS was administered to determine the optimal stimulation site by searching likely regions for the optimal site and the resting motor threshold (RMT) for the right first dorsal interosseous (FDI) muscle [55–57] for a later main experiment using SPS. The RMT was defined as the lowest intensity that evoked a small response (> 50 μV) in 5 or more (typically 5 or 6) of 10 consecutive trials in the relaxed FDI. Motor evoked potentials were recorded from the right FDI muscle using Ag/AgCl sheet electrodes placed over the muscle belly (active) and the metacarpophalangeal joint of the index finger (reference). The signals were sent to an amplifier (The Nihon Kohden Co., Tokyo, Japan) through filters set at 150 Hz to 3 kHz. Biphasic SPS was also conducted in a similar way to determine the optimal stimulation site and the active motor threshold (AMT) for the right FDI muscle for a later main experiment using TBS. The AMT was defined as the lowest intensity that evoked a small response (> 100 μV) in more than 5 of 10 consecutive trials when subjects maintained a slight contraction of the right FDI (10% of the maximum voluntary contraction determined after several trials). The intensity of TMS was not adjusted [58–60] because the aim of the present study is to measure discomfort caused by stimulation of the nerves and muscles outside the brain rather than to investigate the effects of TMS on the brain and behavioral performance.

We used SPS in Blocks 1, 2 and 3 and TBS in Block 4 (Fig 1A). For TBS, 50 Hz triple-pulses were repeated at 5 Hz only for one second instead of the standard 40 seconds [2, 51] to minimize the total amount of stimulation at 11 stimulation positions. Four of the subjects were first examined for 1 second, 2 seconds and 40 seconds, and it was determined that the scores using 1 second were sufficiently similar to those using 40 seconds. The stimulation intensity in Block 1 was set to 30% of the maximum output of the stimulator (a sufficiently small intensity used in TMS studies), while that in Block 2 was set to 50% [50, 61]. In Blocks 3 and 4, the intensity was set individually at 120% RMT and 80% AMT of each subject. The monophasic RMT was 48.0 ± 6.8% (mean ± SD) of the maximum stimulator output, and the biphasic AMT was 35.8 ± 3.4%. After Block 4, in 10 of the 20 subjects who had sufficient additional time for the retest, one block was additionally administered as a retest of Block 2 using the same intensity (50%) as that in Block 2.

The scalp locations of the stimulation were measured and marked using the extended 10–20 system designed for electroencephalogram examination. Stimulations were administered to 11 electrode locations in the extended 10–20 system covering the prefrontal cortex of the right hemisphere, which implements response inhibition [56, 57, 62, 63] that we investigate using the present results (Fig 1B). In each block of the main experiments, stimulations were administered to each location in the predetermined order (Fp2, AF4, AF8, F8, F6, F4, F2, FC2, FC4, FC6 and FC8) for one-half of the subjects and in the reverse order for the other half of the subjects.

### Evaluation scores

After each trial of SPS and TBS, the subjects were instructed to report the extent of discomfort they felt from the stimulation and the extent to which they could tolerate it (Fig 1C). Discomfort was evaluated using a numeric rating scale similar to the visual analogue score with a range from 0 to 10, where 0 represented that there was no discomfort at all, and 10 represented that discomfort was maximally severe to “complete the experiment.” In SPS, to “complete the experiment” meant to receive the stimulation every 30 s for one hour. In TBS, to “complete the experiment” meant to receive the stimulation continuously for 40 seconds, similar to the original protocol [2, 51]. The tolerability was evaluated using a three-level descriptive scale (“none,” “moderate,” and “severe”), in which “none” represented that no feeling of intolerance occurred, “moderate” represented that the stimulation was sensible but tolerable, and “severe” represented that the stimulation was intolerable and should be stopped immediately.

### Online navigation

An online navigation system (TMS Navigator-SW, Localite GmbH, Germany) was used to enssure that the stimulation was administered at the accurate locations. The image data used for navigation were acquired using a 3-T MRI scanner and a 64-channel head coil (Siemens Prisma, Erlanger, Germany). T1-weighted structural images were obtained for anatomical reference (resolution = 0.8 × 0.8 × 0.8 mm^3^). The T1 images were registered to the subject’s head in space using a tracking device and navigation software. The position and orientation of the TMS coil were also registered to the subject’s head in space and were recorded during each time of stimulation.

### Data analysis

Discomfort ratings in the dorsolateral vs. ventrolateral prefrontal cortex were tested using a paired t-test at a group level. The test-retest reproducibility of the discomfort ratings was examined using a correlation of the ratings across electrode positions at the test and retest for each subject, and the correlation coefficients were transformed to Fisher’s z and were tested using a paired t-test at a group level. The test-retest reproducibility of the intolerability ratings was examined by calculating the percentage of the test-retest matches for each subject and was evaluated using a nonparametric test (Wilcoxon signed-rank test) at a group level. Logistic regression analyses were performed to investigate the relationship between the discomfort rating (0 to 10) as an independent variable and the proportion of intolerability (“none,” “moderate,” or “severe”) as a dependent variable.

### Interpolation to generate 3D probability maps

It would be helpful to predict the level of discomfort and tolerability at MNI coordinates of the voxels of interest in the lateral prefrontal cortex. We interpolated the evaluation scores collected at 11 stimulation positions and calculated the evaluation scores at all coordinates in the lateral prefrontal cortex. For the interpolation processing in a three-dimensional space, a stimulation line was defined. The stimulation line indicates the geometrical vector between the TMS coil and the target brain region, and the same discomfort and intolerability ratings were assigned along the stimulation line for each stimulation position, stimulus condition and subject. The predicted value V_predict_, of the discomfort or the intolerability was calculated for each voxel in the lateral prefrontal cortex by interpolating the values from the three nearest stimulation lines as follows:

$$V_{predict}=V_{1}\frac{d_{2}+d_{3}}{2{(d}_{1}+d_{2}+d_{3})}+V_{2}\frac{d_{3}+d_{1}}{2{(d}_{1}+d_{2}+d_{3})}+V_{3}\frac{d_{1}+d_{2}}{2{(d}_{1}+d_{2}+d_{3})}$$

where V_1_, V_2_, and V_3_ are the original values of the 1st, 2nd, and 3rd nearest stimulation lines, respectively, and d_1_, d_2_, and d_3_ are the distances between the voxel and the 1st, 2nd, and 3rd lines, respectively. The maps of the predicted values calculated for each subject were normalized in the standard MNI space (ICBM152) and were averaged across subjects. Thus, the rating of discomfort could be predicted for each specific voxel, with a range from 0 to 10. Intolerability reports were transformed into binary values in the interpolation processing: “none” (no feeling of intolerance) or “moderate” (the stimulation was sensible but tolerable) as 0 and “severe” (the stimulation was intolerable and should be stopped immediately) as 1. Thus, the probability of a stimulation being intolerable at a voxel (0 to 1) could be predicted.

## Results

### Discomfort and intolerability

**Fig 2:**
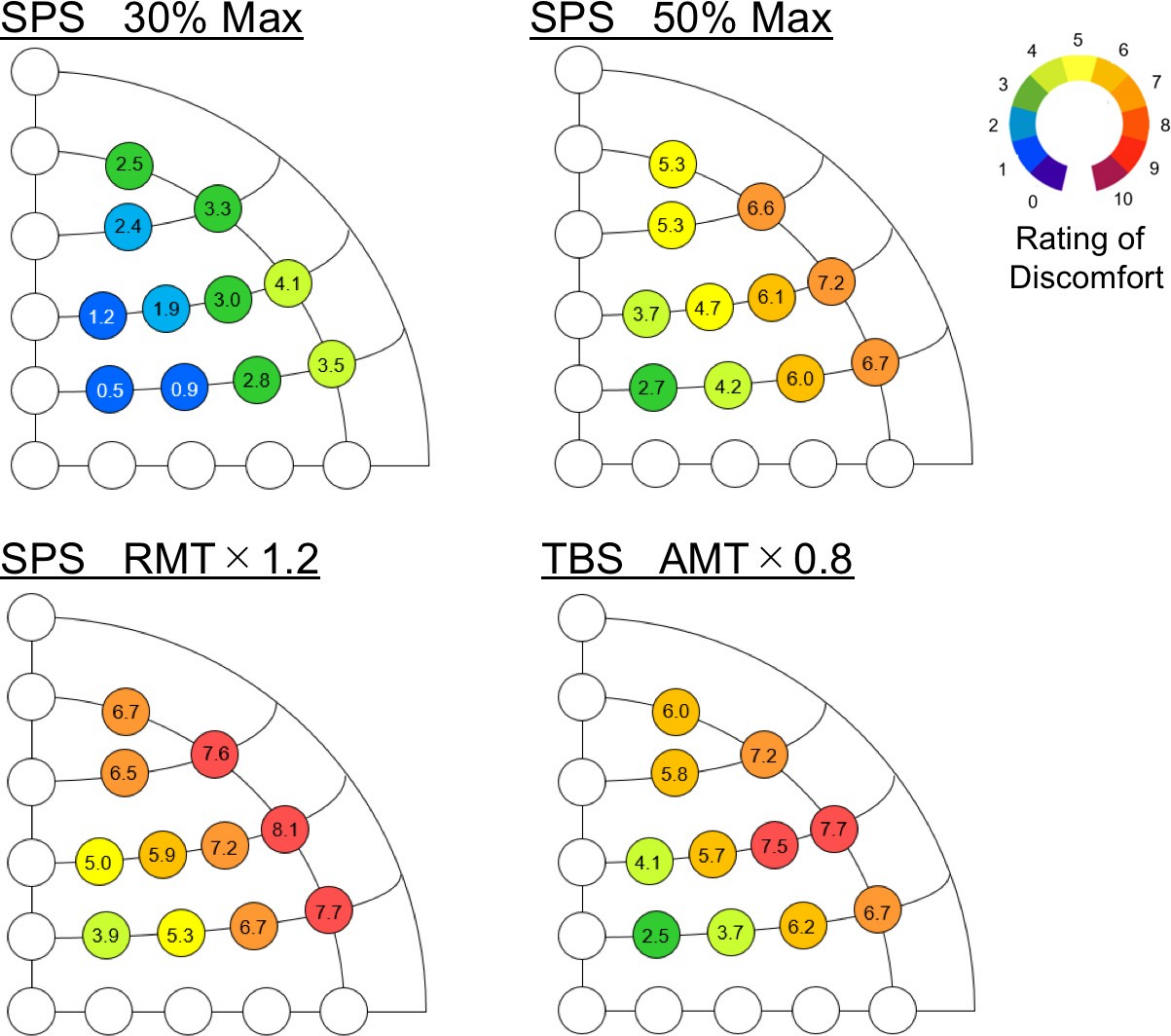
Discomfort rating at 11 stimulation positions. The stimulation conditions consisted of SPS at 30% of the maximal output (upper left), SPS at 50% of the maximal output (upper right), SPS at 120% of the RMT (bottom left), and TBS at 80% of the AMT (bottom right). The color scale indicates the degree of discomfort averaged for each electrode across subjects (0: minimal and 10: maximal for each subject).

**Fig 3:**
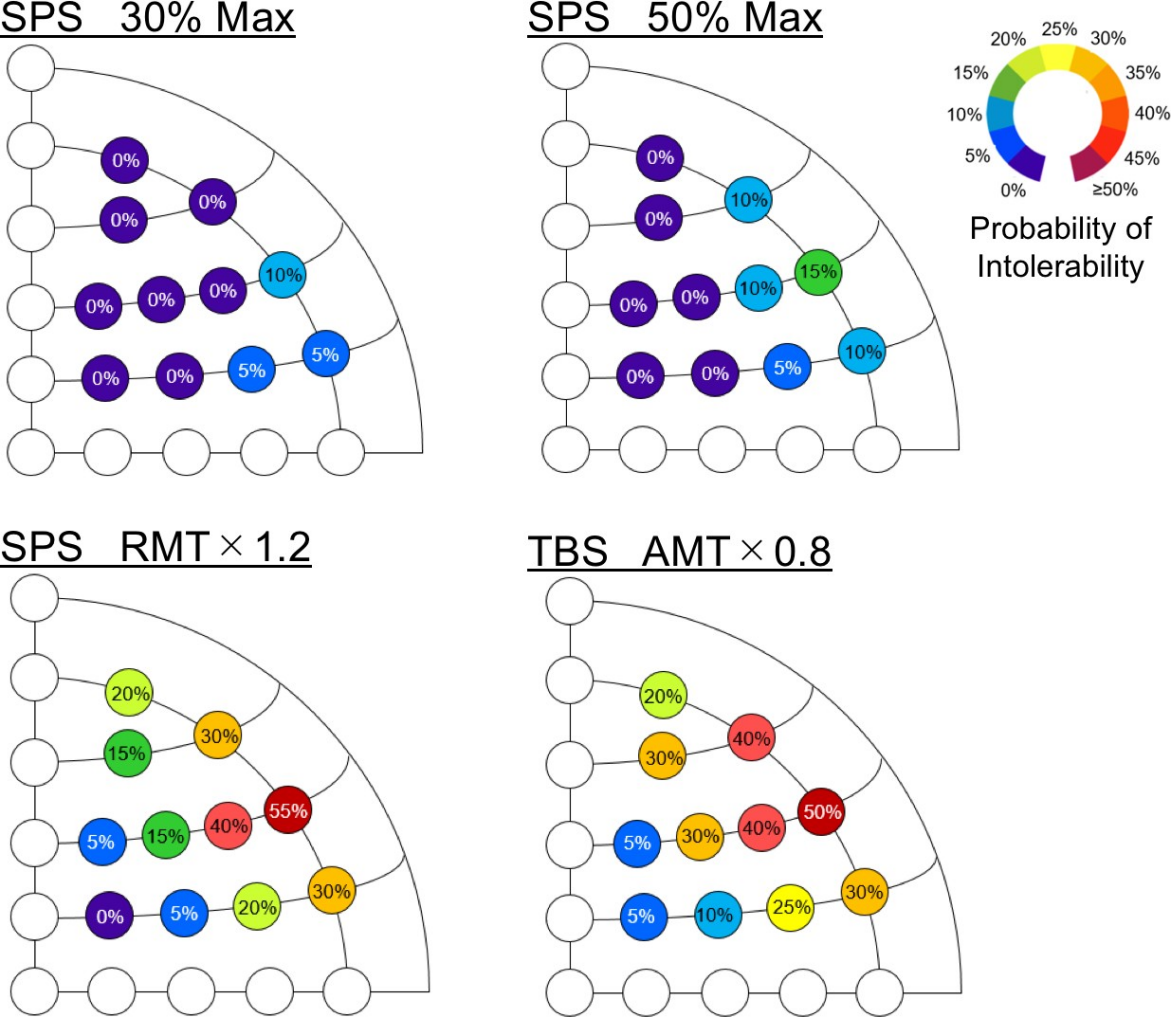
Percentage intolerability at 11 stimulation positions. The stimulation conditions consisted of SPS at 30% of the maximal output (upper left), SPS at 50% of the maximal output (upper right), SPS at 120% of the RMT (bottom left), and TBS at 80% of the AMT (bottom right). The color scale indicates the probability of intolerability (0: tolerable and 1: intolerable for each subject).

The ratings of discomfort induced by SPS and TBS were averaged across subjects for each stimulation position (Fig 2). In the SPS blocks (Blocks 1, 2 and 3), the overall rating increased with the stimulus intensity. The rating was relatively greater in the ventrolateral prefrontal cortex (Fp2, AF8, F8 and FC8) than that in the dorsolateral prefrontal cortex (F2, F4, FC2 and FC4) [t(19) = 8.6, P < 0.001], which is consistent with the smaller number of the extant TMS studies of the ventrolateral prefrontal cortex (approximately one-third, based on our PubMed search). S1 Fig shows the ratio of the increase in the TMS intensity to the level of discomfort and tolerability based on the results of 30% and 50% maximal output in SPS. In the TBS block (Block 4), the rating of discomfort was similar to Block 2 both in magnitude and spatial pattern [t(19) = 6.8, P < 0.001]. Both SPS and TBS elicited maximal discomfort at the stimulation position F8. Fig 3 shows how the discomfort was intolerable for the subjects. The spatial pattern of intolerability was similar to that of the discomfort rating in each stimulus condition, and SPS and TBS at F8 under the standard TMS protocol (SPS: 120% of RMT; TBS: 80% of AMT) were intolerable for approximately 10 of 20 subjects.

### Test-retest reliability

**Fig 4:**
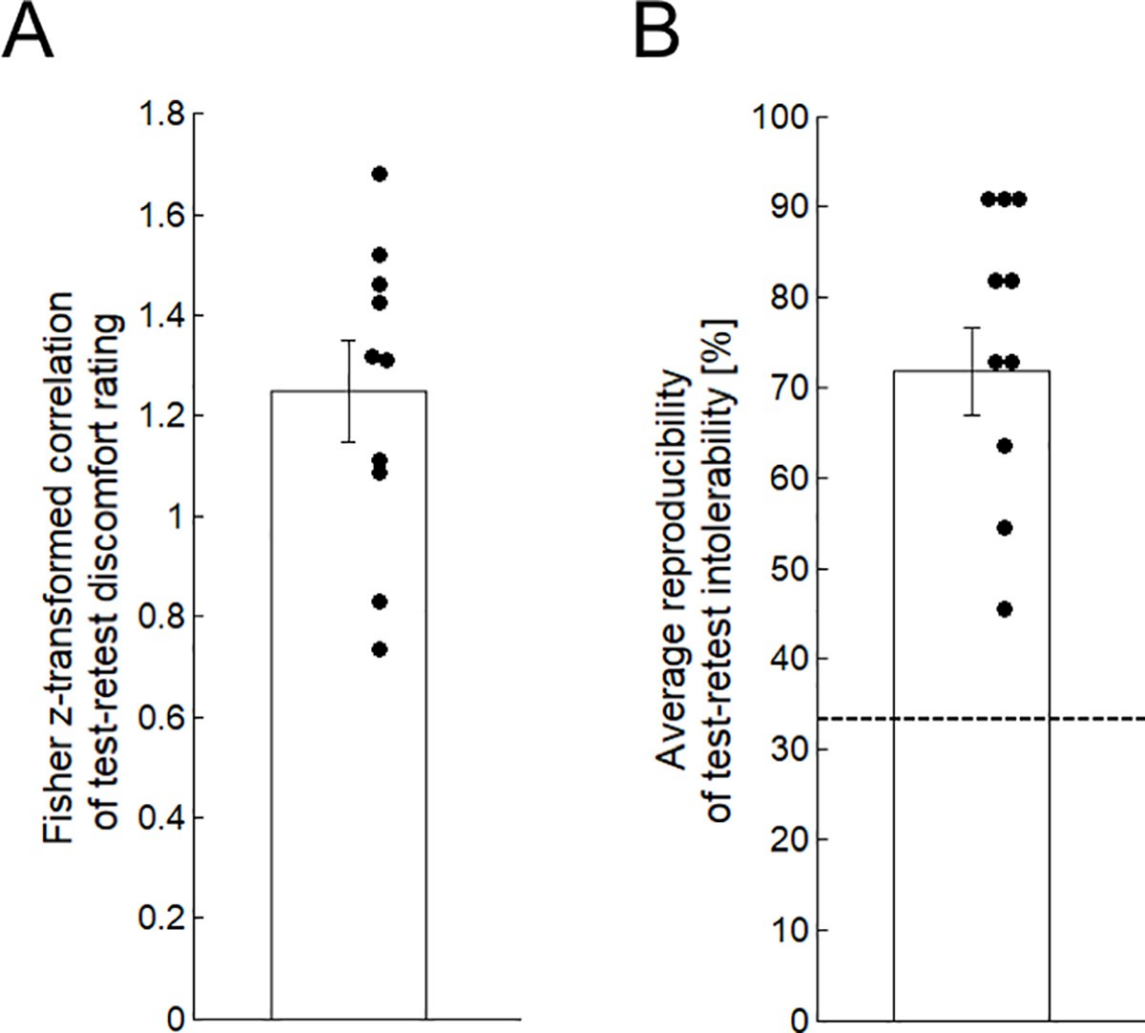
Test-retest reliability. (A) The correlation (Fisher’s z) between two stimulation blocks under SPS at 50% of the maximal output. The bar graph indicates the standard errors of the means, and one point indicates each subject. (B) The average reproducibility of the three ranks of intolerability (“none,” “moderate,” and “severe”). The dotted line indicates the chance level (33%).

To examine the test-retest reliability, we calculated the Pearson’s correlation coefficient of the discomfort ratings for each subject between the test (Block 2) and retest blocks at the 11 stimulation positions. The ratings of discomfort of the test blocks significantly correlated with those of the retest blocks [Fisher’s z: mean = 1.25, SD = 0.30, one-sample t-test, t(9) = 13.0, P < 0.001] (Fig 4A). We also examined the consistency of the tolerability between the test and retest blocks for each subject at the 11 stimulation positions. The subjects consistently reported their intolerability [mean = 71.8%, SD = 15.1%, Wilcoxon signed-rank test, z = 2.8, P = 0.005, chance-level = 33.3%] (Fig 4B). These results indicate that discomfort and tolerability were consistent across the different blocks.

### Relationship between the two evaluation scores

**Fig 5:**
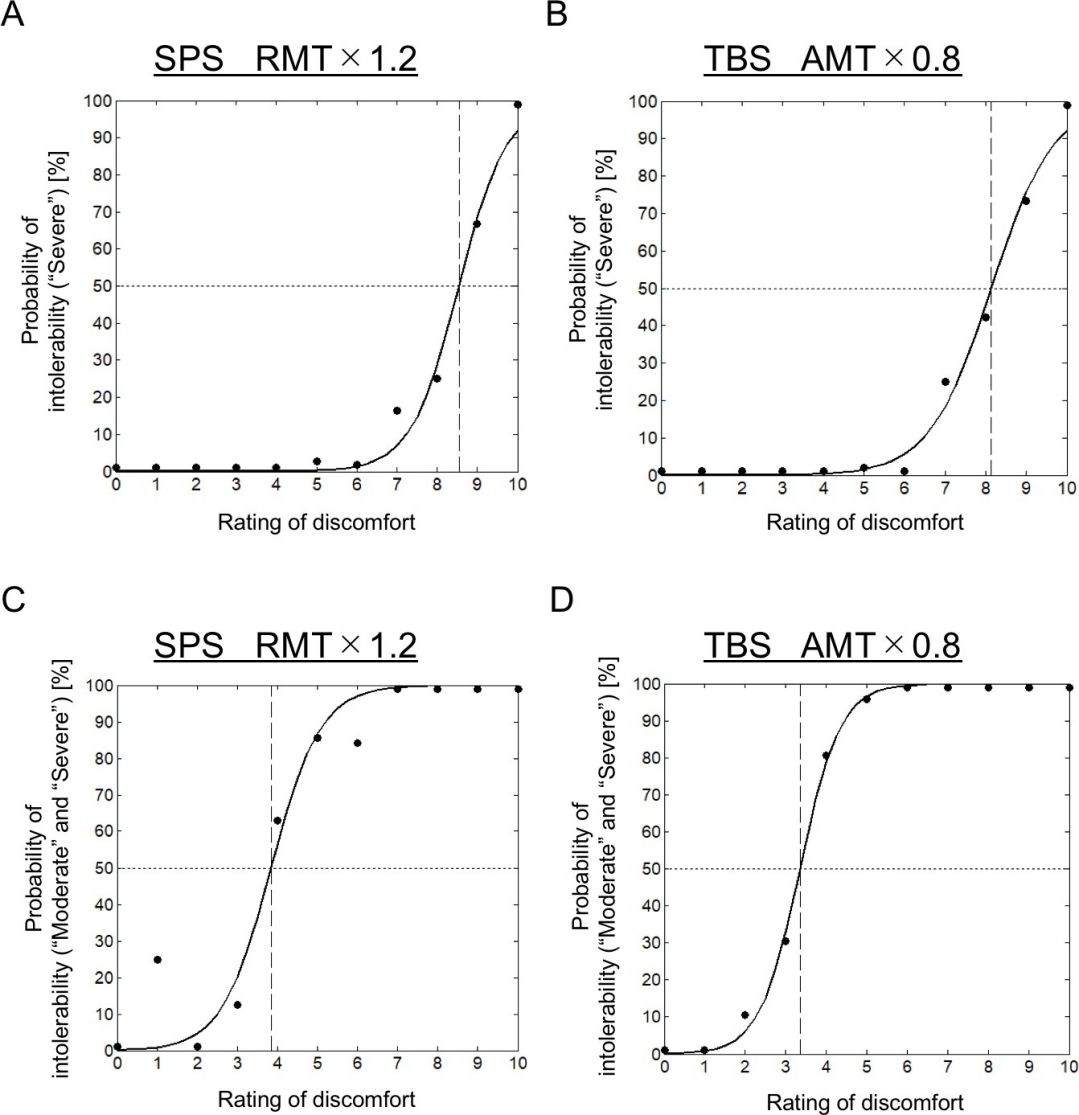
Relationship between discomfort and intolerability. (A) The relationship between the discomfort rating (0 to 10) as an independent variable and the proportion of intolerability (“none,” “moderate,” or “severe”) as a dependent variable. The solid curve indicates a logistic regression. The probability of “severe” for each rating value in the stimulation positions of the subjects was calculated for the SPS at 120% of the RMT. (B) Similar to (A), with the exception that the probability of “severe” was calculated for the TBS at 80% of the AMT. (C) Similar to (A), with the exception that the probability of “severe” or “moderate” for each rating value in the stimulation positions of the subjects was calculated for the SPS at 120% of the RMT. (D) Similar to (A), with the exception that the probability of “severe” or “moderate” was also calculated for the TBS at 80% of the AMT.

As shown in Figs 2 and 3, the discomfort and intolerability ratings were closely related. We used a logistic regression analysis to quantify the relationship between the discomfort rating (0 to 10) and the proportion of intolerability (“none,” “moderate,” or “severe”). The probability of “severe” for each rating value in the 11 stimulation positions of the subjects was calculated for the SPS at 120% of the RMT, and the 50% point scored 8.55 in the discomfort rating (Fig 5A). The probability of “severe” was also calculated for the TBS at 80% of the AMT, and the 50% point scored 8.16 in the discomfort rating (Fig 5B), consistent with the results of the SPS. Similarly, the probability of “severe” or “moderate,” rather than “severe” only, for each rating value in the 11 stimulation positions of the subjects was subsequently calculated for the SPS at 120% of the RMT, and the 50% point scored 3.84 in the discomfort rating (Fig 5C). The probability of “severe” or “moderate” was also calculated for the TBS at 80% of the AMT, and the 50% point scored 3.36 in the discomfort rating (Fig 5D), consistent with the results of the SPS.

### Estimation of discomfort and intolerability in any lateral prefrontal region

**Fig 6:**
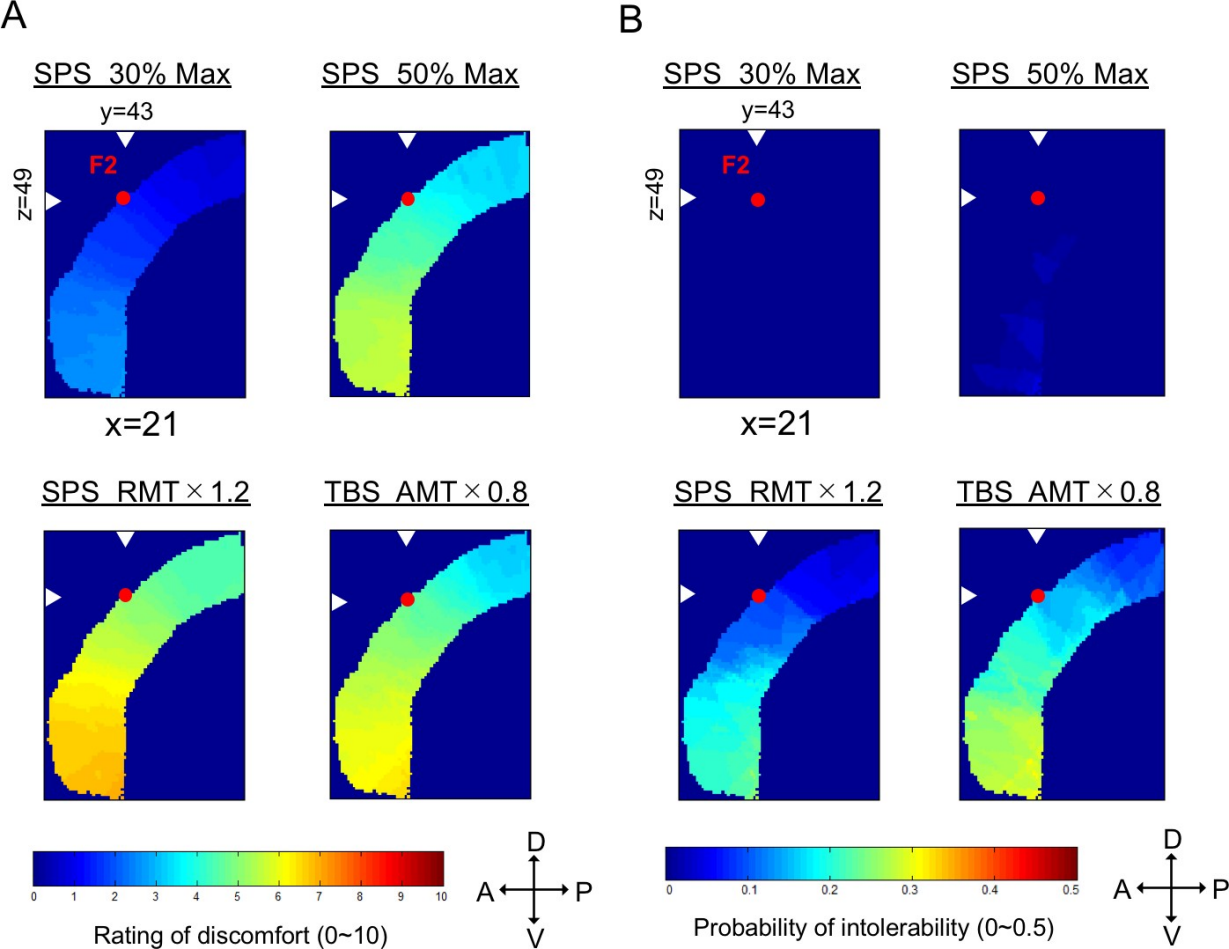
Brain mapping of discomfort and intolerability. (A) Sagittal sections of discomfort mapping under the four stimulation conditions. The images were calculated 4 cm from the head surface for convenience. The color scale indicates the level of discomfort. D: dorsal, V: ventral, A: anterior, and P: posterior. (B) Sagittal sections of the intolerability mapping under the four stimulation conditions. The color scale indicates the probability of intolerability.

The two evaluation scores were measured only at the 11 stimulation positions. To estimate the value of the two evaluation scores at any target voxel in the lateral prefrontal cortex, the values at the voxel were interpolated from the three nearest stimulation lines (refer to Methods). Table 1 shows the average MNI coordinates of the intersection points where the 11 stimulation lines cross the brain surface. Fig 6A shows one example slice (sagittal slice at X = 21) of such images of the discomfort rating near the electrode F2 for the four stimulation conditions. The slice is also shown for the intolerability probability in Fig 6B. Because the extent of stimulation into the brain tissue is limited, the images were calculated only for 4 cm from the head surface to cover the brain surface region. These interpolation images enable us to estimate the additional number of subjects required in stimulating a target in the lateral prefrontal cortex. The image files for the interpolated values are available as supplementary materials (S1 File).

**Table 1:** Coordinates of the brain surface that correspond to the stimulation positions.

Electrode	MNI coordinates (mm) (mean ± SD)		
	X	Y	Z
FP2	27.1 ± 4.5	67.4 ± 2.2	-3.8 ± 6.2
AF4	28.0 ± 4.3	63.7 ± 3.5	17.6 ± 9.0
AF8	46.6 ± 3.6	53.8 ± 3.5	-6.1 ± 4.9
F2	20.6 ± 5.8	43.4 ± 6.6	48.8 ± 7.9
F4	41.3 ± 4.3	42.5 ± 6.5	35.2 ± 9.4
F6	54.1 ± 3.0	39.0 ± 6.2	13.7 ± 8.3
F8	55.4 ± 3.9	29.1 ± 3.7	-10.9 ± 7.6
FC2	27.6 ± 6.6	13.4 ± 7.5	65.6 ± 3.9
FC4	51.2 ± 4.8	12.8 ± 9.6	48.7 ± 7.7
FC6	64.5 ± 2.7	9.8 ± 9.2	20.6 ± 9.9
FC8	64.0 ± 3.5	6.3 ± 5.8	-13.9 ± 7.5

## Discussion

The present study investigated discomfort and intolerability for subjects during SPS and TBS administered to the lateral prefrontal stimulation positions of the high-resolution extended 10–20 system. The SPS and TBS of the ventrolateral prefrontal cortex elicited greater discomfort and intolerability than that of the dorsolateral prefrontal cortex. The magnitude of discomfort and intolerability was similar in the SPS and TBS, and the intolerability was maximal (~ 50%) at stimulation position F8, which indicates that the number of subjects to be recruited would have to be double the intended sample size. The image file of the voxelwise probability of intolerability in the lateral prefrontal cortex is provided as supplementary data (S1 File), which would be useful in estimating the additional number of subjects to be recruited.

Discomfort during TMS to the lateral prefrontal cortex consists of pain and twitches caused by nerves and muscles, respectively [9–16, 49, 50]. The first branch of the trigeminal nerve (ophthalmic nerve) innervates a wide range of the forehead. TMS of the rostral part of the lateral prefrontal cortex often activates the trigeminal nerve; however, the stimulation of the ventrolateral prefrontal cortex may cause greater discomfort due to the activation of a greater number of nerve fibers. Moreover, the temporal muscle is activated by stimulation of the caudal part of the ventrolateral prefrontal cortex. The spatial pattern of discomfort reported in this study, in which greater discomfort was reported in the ventrolateral prefrontal cortex in all four stimulation conditions, 30% Max SPS, 50% Max SPS, 120% RMT SPS and TBS, appears consistent with the sum of the distribution of the involved nerves and muscles.

TBS was administered for only 1 second in the present study to minimize the total amount of stimulation over the 11 stimulation positions instead of the 40 seconds of the standard protocol [2, 51]. It is possible that the stimulation effect accumulates over the 40 seconds, making the intolerability greater than the reported results. On the other hand, subjects tend to become habituated over time [43, 45], and it seems likely that the increase of intolerability would be minimal. It is noted that the present study examined Japanese subjects. The motor threshold is known to be lower in Caucasian than in Han Chinese subjects [64], and intolerability is likely lower in Caucasian subjects because of the lower RMT and AMT. It is also noted that the effect of TMS depends on the distance between TMS coil and the target brain region, and a greater stimulation intensity will be required when deeper brain regions are targeted [58–60]. Therefore, the present results of discomfort/intolerability primarily pertain to surface regions.

The required number of subjects for inclusion in TMS studies depends on the significance level, the statistical power, and the magnitude of expected changes in measured behavioral/MEP effects induced by TMS. Intolerability measured in the present study can be used to expect potential drop-outs due to discomfort experienced during TMS and calculate the likely number of subjects to be recruited in the initial phase of the study. This approach can be applied to other forms of brain stimulation techniques, such as transcranial current direction stimulation [65–67]. The interpolation images (Fig 6) enable us to estimate the additional number of subjects required in stimulating activated regions in the lateral prefrontal cortex reported in previous fMRI studies. For example, in the study by Osada et al., 2019 [57], for the lateral prefrontal activation located at MNI coordinates (49, 11, 26), the estimated probability of intolerability for the region was 18% and 25% for the SPS and TBS, respectively. The results indicate that when 20 subjects are required for data collection, the actual number of subjects to be recruited would be approximately 25. In addition, in the study by Chikazoe et al., 2009 [68], for the lateral prefrontal activation located at (56, 16, 16), the estimated probability of intolerability was 30% and 32% for the SPS and TBS experiments, respectively, which indicates that when the data are to be collected from 20 subjects, the actual number of subjects to be recruited would be approximately 30 (for example, 30% of 30 subjects, i.e., 9 subjects, will be excluded, resulting in a final size of 21, approximately 20, subjects).

Importantly, even when stimulation of the ventrolateral prefrontal cortex is experimentally executable, the discomfort caused by stimulation confounds the behavioral performance [50]. For the confound to be managed, it is advisable to choose control sites with similar levels of discomfort rather than simply using sham TMS [49, 69–72]. Although TMS studies of the ventrolateral prefrontal cortex are relatively rare at this point, the present study may encourage future studies targeting the ventrolateral prefrontal cortex by providing the additional number of subjects required for the study in planning the experimental protocols.

## Supporting information

S1 Fig The ratio of the increase in the TMS intensity to the level of discomfort and tolerability based on the results of 30% and 50% maximal output in SPS. (TIFF)

S1 Dataset The ratings of discomfort and intolerability in each stimulation condition at each electrode position for each subject. (XLSX)

S1 File The image files for the interpolated values of discomfort and intolerability in each stimulation condition. (ZIP)

## References

[1] Pascual-Leone A, Walsh V, Rothwell J. Transcranial magnetic stimulation in cognitive neuroscience—virtual lesion, chronometry, and functional connectivity. Curr Opin Neurobiol. 2000;10(2):232–7. 10753803 10.1016/s0959-4388(00)00081-7

[2] Huang YZ, Edwards MJ, Rounis E, Bhatia KP, Rothwell JC. Theta burst stimulation of the human motor cortex. Neuron. 2005;45(2):201–6. 10.1016/j.neuron.2004.12.033 15664172

[3] Paus T. Inferring causality in brain images: a perturbation approach. Philos Trans R Soc Lond B Biol Sci. 2005;360(1457):1109–14. 10.1098/rstb.2005.1652 16087451 PMC1854935

[4] Hallett M. Transcranial magnetic stimulation: a primer. Neuron. 2007;55(2):187–99. 10.1016/j.neuron.2007.06.026 17640522

[5] Narayana S, Papanicolaou AC, McGregor A, Boop FA, Wheless JW. Clinical Applications of Transcranial Magnetic Stimulation in Pediatric Neurology. J Child Neurol. 2015;30(9):1111–24. 10.1177/0883073814553274 25342309

[6] Parkin BL, Ekhtiari H, Walsh VF. Non-invasive Human Brain Stimulation in Cognitive Neuroscience: A Primer. Neuron. 2015;87(5):932–45. 10.1016/j.neuron.2015.07.032 26335641

[7] Rossini PM, Burke D, Chen R, Cohen LG, Daskalakis Z, Di Iorio R, et al Non-invasive electrical and magnetic stimulation of the brain, spinal cord, roots and peripheral nerves: Basic principles and procedures for routine clinical and research application. An updated report from an I.F.C.N. Committee. Clin Neurophysiol. 2015;126(6):1071–107. 10.1016/j.clinph.2015.02.001 25797650 PMC6350257

[8] Miyashita Y. The cutting edge in brain science and sportology. Juntendo Medical Journal. 2016;62:6–11.

[9] Wassermann EM. Risk and safety of repetitive transcranial magnetic stimulation: report and suggested guidelines from the International Workshop on the Safety of Repetitive Transcranial Magnetic Stimulation, June 5–7, 1996. Electroencephalogr Clin Neurophysiol. 1998;108(1):1–16. 9474057 10.1016/s0168-5597(97)00096-8

[10] Abler B, Walter H, Wunderlich A, Grothe J, Schönfeldt-Lecuona C, Spitzer M, et al Side effects of transcranial magnetic stimulation biased task performance in a cognitive neuroscience study. Brain Topogr. 2005;17(4):193–6. 16110769 10.1007/s10548-005-6028-y

[11] Grossheinrich N, Rau A, Pogarell O, Hennig-Fast K, Reinl M, Karch S, et al Theta burst stimulation of the prefrontal cortex: safety and impact on cognition, mood, and resting electroencephalogram. Biol Psychiatry. 2009;65(9):778–84. 10.1016/j.biopsych.2008.10.029 19070834

[12] Poreisz C, Paulus W, Moser T, Lang N. Does a single session of theta-burst transcranial magnetic stimulation of inferior temporal cortex affect tinnitus perception?. BMC Neurosci. 2009;10:54 10.1186/1471-2202-10-54 19480651 PMC2703646

[13] Rossi S, Hallett M, Rossini PM, Pascual-Leone A. Safety, ethical considerations, and application guidelines for the use of transcranial magnetic stimulation in clinical practice and research. Clin Neurophysiol. 2009;120(12):2008–39. 10.1016/j.clinph.2009.08.016 19833552 PMC3260536

[14] Oberman L, Edwards D, Eldaief M, Pascual-Leone A. Safety of theta burst transcranial magnetic stimulation: a systematic review of the literature. J Clin Neurophysiol. 2011;28(1):67–74. 10.1097/WNP.0b013e318205135f 21221011 PMC3260517

[15] Tarapore PE, Picht T, Bulubas L, Shin Y, Kulchytska N, Meyer B, et al Safety and tolerability of navigated TMS for preoperative mapping in neurosurgical patients. Clin Neurophysiol. 2016;127(3):1895–900. 10.1016/j.clinph.2015.11.042 26762952

[16] Peterchev AV, Luber B, Westin GG, Lisanby SH. Pulse Width Affects Scalp Sensation of Transcranial Magnetic Stimulation. Brain Stimul. 2017;10(1):99–105. 10.1016/j.brs.2016.09.007 28029593 PMC5241181

[17] Machii K, Cohen D, Ramos-Estebanez C, Pascual-Leone A. Safety of rTMS to non-motor cortical areas in healthy participants and patients. Clin Neurophysiol. 2006;117(2):455–71. 10.1016/j.clinph.2005.10.014 16387549

[18] Loo CK, McFarquhar TF, Mitchell PB. A review of the safety of repetitive transcranial magnetic stimulation as a clinical treatment for depression. Int J Neuropsychopharmacol. 2008;11(1):131–47. 10.1017/S1461145707007717 17880752

[19] Maizey L, Allen CP, Dervinis M, Verbruggen F, Varnava A, Kozlov M, et al Comparative incidence rates of mild adverse effects to transcranial magnetic stimulation. Clin Neurophysiol. 2013;124(3):536–44. 10.1016/j.clinph.2012.07.024 22986284

[20] Ro T, Henik A, Machado L, Rafal RD. Transcranial magnetic stimulation of the prefrontal cortex delays contralateral endogenous saccades. J Cogn Neurosci. 1997;9(4):433–40. 10.1162/jocn.1997.9.4.433 23968209

[21] Müri RM, Gaymard B, Rivaud S, Vermersch A, Hess CW, Pierrot-Deseilligny C. Hemispheric asymmetry in cortical control of memory-guided saccades. A transcranial magnetic stimulation study. Neuropsychologia. 2000;38(8):1105–11. 10838145 10.1016/s0028-3932(00)00030-0

[22] Mull BR, Seyal M. Transcranial magnetic stimulation of left prefrontal cortex impairs working memory. Clin Neurophysiol. 2001;112(9):1672–5. 11514250 10.1016/s1388-2457(01)00606-x

[23] Mottaghy FM, Gangitano M, Krause BJ, Pascual-Leone A. Chronometry of parietal and prefrontal activations in verbal working memory revealed by transcranial magnetic stimulation. Neuroimage. 2003;18(3):565–75. 12667834 10.1016/s1053-8119(03)00010-7

[24] Nyffeler T, Pierrot-Deseilligny C, Pflugshaupt T, von Wartburg R, Hess CW, Müri RM. Information processing in long delay memory-guided saccades: further insights from TMS. Exp Brain Res. 2004;154(1):109–12. 10.1007/s00221-003-1663-6 14578999

[25] Morishima Y, Akaishi R, Yamada Y, Okuda J, Toma K, Sakai K. Task-specific signal transmission from prefrontal cortex in visual selective attention. Nat Neurosci. 2009;12(1):85–91. 10.1038/nn.2237 19098905

[26] Basso D, Ferrari M, Palladino P. Prospective memory and working memory: asymmetrical effects during frontal lobe TMS stimulation. Neuropsychologia. 2010;48(11):3282–90. 10.1016/j.neuropsychologia.2010.07.011 20637788

[27] Ott DVM, Ullsperger M, Jocham G, Neumann J, Klein TA. Continuous theta-burst stimulation (cTBS) over the lateral prefrontal cortex alters reinforcement learning bias. Neuroimage. 2011;57(2):617–23. 10.1016/j.neuroimage.2011.04.038 21554966

[28] D'Ardenne K, Eshel N, Luka J, Lenartowicz A, Nystrom LE, Cohen JD. Role of prefrontal cortex and the midbrain dopamine system in working memory updating. Proc Natl Acad Sci USA. 2012;109(49):19900–9. 10.1073/pnas.1116727109 23086162 PMC3523834

[29] Duque J, Labruna L, Verset S, Olivier E, Ivry RB. Dissociating the role of prefrontal and premotor cortices in controlling inhibitory mechanisms during motor preparation. J Neurosci. 2012;32(3):806–16. 10.1523/JNEUROSCI.4299-12.2012 22262879 PMC3304578

[30] Bilek E, Schafer A, Ochs E, Esslinger C, Zangl M, Plichta MM, et al Application of high-frequency repetitive transcranial magnetic stimulation to the DLPFC alters human prefrontal-hippocampal functional interaction. J Neurosci. 2013;33(16):7050–6. 10.1523/JNEUROSCI.3081-12.2013 23595762 PMC6618883

[31] Gratton C, Lee TG, Nomura EM, D'Esposito M. The effect of theta-burst TMS on cognitive control networks measured with resting state fMRI. Front Syst Neurosci. 2013;7:124 10.3389/fnsys.2013.00124 24416003 PMC3874542

[32] Hartwigsen G, Saur D, Price CJ, Ulmer S, Baumgaertner A, Siebner HR. Perturbation of the left inferior frontal gyrus triggers adaptive plasticity in the right homologous area during speech production. Proc Natl Acad Sci USA. 2013;110(41):16402–7. 10.1073/pnas.1310190110 24062469 PMC3799383

[33] Smittenaar P, FitzGerald TH, Romei V, Wright ND, Dolan RJ. Disruption of dorsolateral prefrontal cortex decreases model-based in favor of model-free control in humans. Neuron. 2013;80(4):914–9. 10.1016/j.neuron.2013.08.009 24206669 PMC3893454

[34] Ubaldi S, Barchiesi G, Cattaneo L. Bottom-up and top-down visuomotor responses to action observation. Cereb Cortex. 2015;25(4):1032–41. 10.1093/cercor/bht295 24132640

[35] Rahnev D, Nee DE, Riddle J, Larson AS, D'Esposito M. Causal evidence for frontal cortex organization for perceptual decision making. Proc Natl Acad Sci USA. 2016;113(21):6059–64. 10.1073/pnas.1522551113 27162349 PMC4889369

[36] Sagliano L, D'Olimpio F, Panico F, Gagliardi S, Trojano L. The role of the dorsolateral prefrontal cortex in early threat processing: a TMS study. Soc Cogn Affect Neurosci. 2016;11(12):1992–8. 10.1093/scan/nsw105 27510494 PMC5141957

[37] Gogulski J, Zetter R, Nyrhinen M, Pertovaara A, Carlson S. Neural Substrate for Metacognitive Accuracy of Tactile Working Memory. Cereb Cortex. 2017;27(11):5343–52. 10.1093/cercor/bhx219 28968804

[38] Smalle E, Panouilleres M, Szmalec A, & Möttönen R. Language learning in the adult brain: disrupting the dorsolateral prefrontal cortex facilitates word-form learning. Scientific reports. 2017;7(1):13966 10.1038/s41598-017-14547-x 29070879 PMC5656634

[39] Chung SW, Rogasch NC, Hoy KE, Sullivan CM, Cash RFH., Fitzgerald PB. Impact of different intensities of intermittent theta burst stimulation on the cortical properties during TMS-EEG and working memory performance. Hum Brain Mapp. 2018;39(2):783–802. 10.1002/hbm.23882 29124791 PMC6866298

[40] Zhao D, Zhou YD, Bodner M, Ku Y. The Causal Role of the Prefrontal Cortex and Somatosensory Cortex in Tactile Working Memory. Cereb Cortex. 2018;28(10):3468–77. 10.1093/cercor/bhx213 28968894

[41] Devlin JT, Matthews PM, Rushworth MF. Semantic processing in the left inferior prefrontal cortex: a combined functional magnetic resonance imaging and transcranial magnetic stimulation study. J Cogn Neurosci. 2003;15(1):71–84. 10.1162/089892903321107837 12590844

[42] Kahn I, Pascual-Leone A, Theoret H, Fregni F, Clark D, Wagner AD. Transient disruption of ventrolateral prefrontal cortex during verbal encoding affects subsequent memory performance. J Neurophysiol. 2005;94(1):688–98. 10.1152/jn.01335.2004 15758048

[43] Anderson BS, Kavanagh K, Borckardt JJ, Nahas ZH, Kose S, Lisanby SH, et al Decreasing procedural pain over time of left prefrontal rTMS for depression: initial results from the open-label phase of a multi-site trial (OPT-TMS). Brain Stimul. 2009;2(2):88–92. 10.1016/j.brs.2008.09.001 20161310 PMC2699309

[44] Verbruggen F, Aron AR, Stevens MA, Chambers CD. Theta burst stimulation dissociates attention and action updating in human inferior frontal cortex. Proc Natl Acad Sci USA. 2010;107(31):13966–71. 10.1073/pnas.1001957107 20631303 PMC2922216

[45] Borckardt JJ, Nahas ZH, Teal J, Lisanby SH, McDonald WM, Avery D, et al The painfulness of active, but not sham, transcranial magnetic stimulation decreases rapidly over time: results from the double-blind phase of the OPT-TMS Trial. Brain Stimul. 2013;6(6):925–8. 10.1016/j.brs.2013.04.009 23769413 PMC3819406

[46] De Dreu CK, Kret ME, Sligte IG. Modulating prefrontal control in humans reveals distinct pathways to competitive success and collective waste. Soc Cogn Affect Neurosci. 2016;11(8):1236–44. 10.1093/scan/nsw045 27036875 PMC4967807

[47] Hartwright CE, Hardwick RM, Apperly IA, Hansen PC. Resting state morphology predicts the effect of theta burst stimulation in false belief reasoning. Hum Brain Mapp. 2016;37(10):3502–14. 10.1002/hbm.23255 27195942 PMC6867310

[48] Ueno T, Meteyard L, Hoffman P, & Murayama K. The Ventral Anterior Temporal Lobe has a Necessary Role in Exception Word Reading. Cereb Cortex. 2018;28(8):3035–45. 10.1093/cercor/bhy131 29878073 PMC6041960

[49] Holmes NP & Meteyard L. Subjective Discomfort of TMS Predicts Reaction Times Differences in Published Studies. Front Psychol. 2018;9:1989 10.3389/fpsyg.2018.01989 30405482 PMC6200894

[50] Meteyard L & Holmes NP. TMS SMART - Scalp mapping of annoyance ratings and twitches caused by Transcranial Magnetic Stimulation. J Neurosci Methods. 2018;299:34–44. 10.1016/j.jneumeth.2018.02.008 29471064

[51] Suppa A, Huang YZ, Funke K, Ridding MC, Cheeran B, Di Lazzaro V, et al Ten Years of Theta Burst Stimulation in Humans: Established Knowledge, Unknowns and Prospects. Brain Stimul. 2016;9(3):323–35. 10.1016/j.brs.2016.01.006 26947241

[52] Chatrian GE, Lettich E, Nelson PL. Modified nomenclature for the "10%" electrode system. J Clin Neurophysiol. 1988;5(2):183–6. 3250964

[53] Jurcak V, Tsuzuki D, Dan I. 10/20, 10/10, and 10/5 systems revisited: their validity as relative head-surface-based positioning systems. Neuroimage. 2007;34(4):1600–11. 10.1016/j.neuroimage.2006.09.024 17207640

[54] Simmons JP, Nelson LD, Simonsohn U. False-positive psychology: undisclosed flexibility in data collection and analysis allows presenting anything as significant. Psychol Sci. 2011;22(11):1359–66. 10.1177/0956797611417632 22006061

[55] Watanabe T, Hanajima R, Shirota Y, Ohminami S, Tsutsumi R, Terao Y, et al Bidirectional effects on interhemispheric resting-state functional connectivity induced by excitatory and inhibitory repetitive transcranial magnetic stimulation. Hum Brain Mapp. 2014;35:1896–905. 10.1002/hbm.22300 23897535 PMC6869044

[56] Watanabe T, Hanajima R, Shirota Y, Tsutsumi R, Shimizu T, Hayashi T, et al Effects of rTMS of pre-supplementary motor area on fronto basal ganglia network activity during stop-signal task. J Neurosci. 2015;35(12):4813–23. 10.1523/JNEUROSCI.3761-14.2015 25810512 PMC6705371

[57] Osada T, Ohta S, Ogawa A, Tanaka M, Suda A, Kamagata K, et al An essential role of the intraparietal sulcus in response inhibition predicted by parcellation-based network. J Neurosci. 2019;39(13):2509–21. 10.1523/JNEUROSCI.2244-18.2019 30692225 PMC6435821

[58] Stokes MG, Chambers CD, Gould IC, Henderson TR, Janko NE, Allen NB, et al Simple metric for scaling motor threshold based on scalp-cortex distance: application to studies using transcranial magnetic stimulation. J Neurophysiol. 2005 12;94(6):4520–7. 10.1152/jn.00067.2005 16135552

[59] Stokes MG, Chambers CD, Gould IC, English T, McNaught E, McDonald O, et al Distance-adjusted motor threshold for transcranial magnetic stimulation. Clin Neurophysiol. 2007;118(7):1617–25. 10.1016/j.clinph.2007.04.004 17524764

[60] Cai W, George JS, Chambers CD, Stokes MG, Verbruggen F, Aron AR. Stimulating deep cortical structures with the batwing coil: how to determine the intensity for transcranial magnetic stimulation using coil-cortex distance. J Neurosci Methods. 2012;204(2):238–41. 10.1016/j.jneumeth.2011.11.020 22138632 PMC3633572

[61] Tamè L & Holmes NP. Involvement of human primary somatosensory cortex in vibrotactile detection depends on task demand. Neuroimage. 2016;138:184–96. 10.1016/j.neuroimage.2016.05.056 27233148

[62] Jimura K, Hirose S, Kunimatsu A, Ohtomo K, Koike Y, Konishi S. Late enhancement of brain-behavior correlations during response inhibition. Neuroscience. 2014;274:383–92. 10.1016/j.neuroscience.2014.05.058 24912028

[63] Yamasaki T, Ogawa A, Osada T, Jimura K, Konishi S. Within-subject correlation analysis to detect functional areas associated with response inhibition. Front Hum Neurosci. 2018;12:208 10.3389/fnhum.2018.00208 29872386 PMC5972214

[64] Yi X, Fisher KM, Lai M, Mansoor K, Bicker R, Baker SN. Differences between Han Chinese and Caucasians in transcranial magnetic stimulation parameters. Exp Brain Res. 2014;232(2):545–53. 10.1007/s00221-013-3763-2 24240390 PMC3901935

[65] Schabrun SM, Burns E, Thapa T, Hodges P. The response of the primary motor cortex to neuromodulation is altered in chronic low back pain: a preliminary study. Pain Med. 2018;19(6):1227–36. 10.1093/pm/pnx168 29016867

[66] Thapa T, Graven-Nielsen T, Chipchase LS, Schabrun SM. Disruption of cortical synaptic homeostasis in individuals with chronic low back pain. Clin Neurophysiol. 2018;129(5):1090–6. 10.1016/j.clinph.2018.01.060 29472134

[67] Thapa T, Schabrun SM. Test-Retest Reliability of Homeostatic Plasticity in the Human Primary Motor Cortex. Neural Plast. 2018;2018:6207508 10.1155/2018/6207508 29983706 PMC6015686

[68] Chikazoe J, Jimura K, Asari T, Yamashita K, Morimoto H, Hirose S, et al Functional dissociation in right inferior frontal cortex during performance of go/no-go task. Cereb Cortex. 2009;19(1):146–52. 10.1093/cercor/bhn065 18445602

[69] Arana AB, Borckardt JJ, Ricci R, Anderson B, Li X, Linder KJ, et al Focal electrical stimulation as a sham control for repetitive transcranial magnetic stimulation: does it truly mimic the cutaneous sensation and pain of active prefrontal repetitive transcranial magnetic stimulation? Brain Stimul. 2008;1(1):44–51. 10.1016/j.brs.2007.08.006 19424459 PMC2678025

[70] Herwig U, Cardenas-Morales L, Connemann BJ, Kammer T, Schönfeldt-Lecuona C. Sham or real—Post hoc estimation of stimulation condition in a randomized transcranial magnetic stimulation trial. Neurosci Lett. 2010;471(1):30–3. 10.1016/j.neulet.2010.01.003 20064587

[71] Duecker F, de Graaf TA, Jacobs C, Sack AT. Time-and task-dependent non-neural effects of real and sham TMS. PLoS One. 2013;8(9):e73813 10.1371/journal.pone.0073813 24040080 PMC3763998

[72] Duecker F & Sack AT. Rethinking the role of sham TMS. Front Psychol. 2015;6:210 10.3389/fpsyg.2015.00210 25767458 PMC4341423

