# Supplementary figures and images for: More subjects are required for ventrolateral than dorsolateral prefrontal TMS because of intolerability and potential drop-out
Source: PLoS One. 2019 Jun 3;14(6):e0217826. doi: 10.1371/journal.pone.0217826 (PMC6546272; doi:10.1371/journal.pone.0217826)

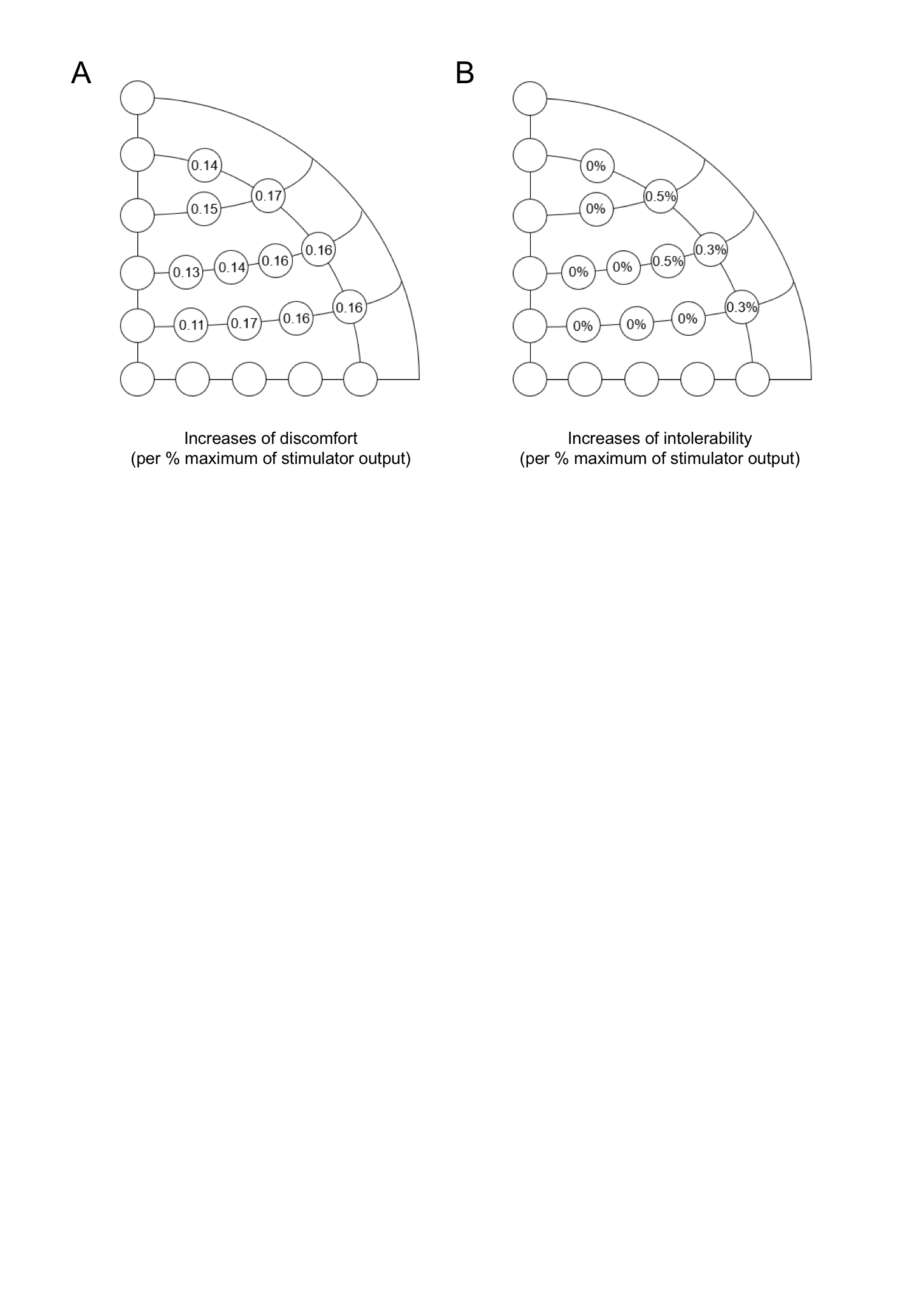

Supplement: S1 Fig — (TIFF) [file pone.0217826.s001.tiff]
